# Enzymatic Degradation Behavior of Self-Degradable Lipase-Embedded Aliphatic and Aromatic Polyesters and Their Blends

**DOI:** 10.1021/acs.biomac.4c00161

**Published:** 2024-06-10

**Authors:** Mario Iván Peñas, Ana Beloqui, Antxon Martínez de Ilarduya, Supakij Suttiruengwong, Rebeca Hernández, Alejandro J. Müller

**Affiliations:** † Institute of Polymer Science and Technology ICTP-CSIC, Juan de la Cierva 3, Madrid 28006, Spain; ‡ Polymat and Department of Polymers and Advanced Materials: Physics, Chemistry and Technology, Faculty of Chemistry, University of the Basque Country UPV/EHU, Paseo Manuel de Lardizabal 3, Donostia-San Sebastián 20018, Spain; § Polymat and Department of Applied Chemistry, Faculty of Chemistry, University of the Basque Country UPV/EHU, Paseo Manuel de Lardizabal 3, Donostia-San Sebastián 20018, Spain; ∥ IKERBASQUE, Basque Foundation for Science, Plaza Euskadi 5, Bilbao 48009, Spain; ⊥ Department of Chemical Engineering, Polytechnic University of Catalonia ETSEIB-UPC, Diagonal 647, Barcelona 08028, Spain; # Sustainable Materials Laboratory, Department of Materials Science and Engineering, Faculty of Engineering and Industrial Technology, Silpakorn University, Nakhon Pathom 73000, Thailand

## Abstract

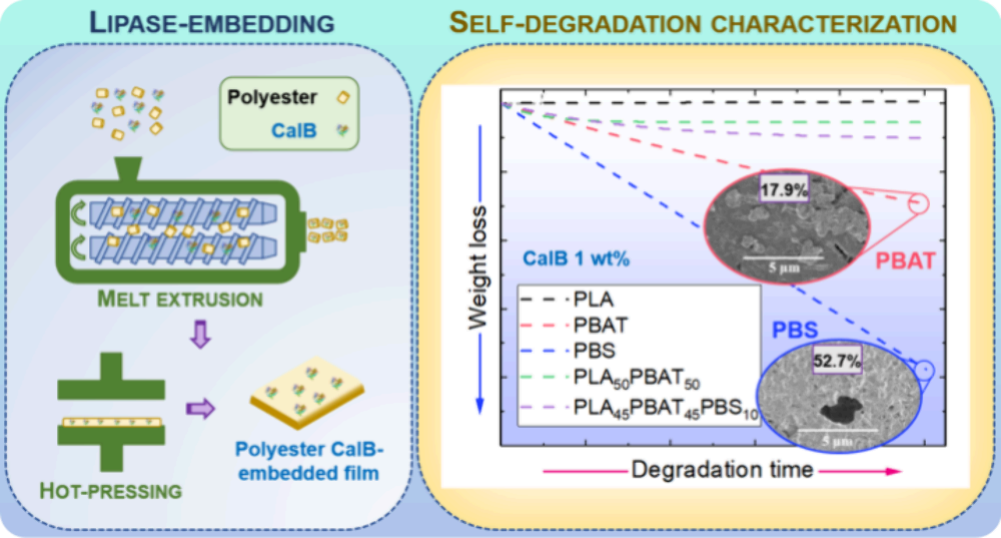

Over the past decade, the preparation of novel materials by enzyme-embedding into biopolyesters has been proposed as a straightforward method to produce self-degrading polymers. This paper reports the preparation and enzymatic degradation of extruded self-degradable films of three different biopolyesters: poly(lactic acid) (PLA), poly(butylene adipate-*co*-terephthalate) (PBAT), and poly(butylene succinate) (PBS), as well as three binary/ternary blends. *Candida antarctica* lipase B (CalB) has been employed for the enzyme-embedding procedure, and to the best of our knowledge, the use of this approach in biopolyester blends has not been reported before. The three homopolymers exhibited differentiated degradation and suggested a preferential attack of CalB on PBS films over PBAT and PLA. Moreover, the self-degradable films obtained from the blends showed slow degradation, probably due to the higher content in PLA and PBAT. These observations pave the way for exploring enzymes capable of degrading all blend components or an enzymatic mixture for blend degradation.

## 1 Introduction

Polyesters, and especially biopolyesters, have attracted a great deal of attention through the past years as natural substitutes to nondegradable fossil-based polymers and a good alternative to common plastics that otherwise end up in landfills or even in the oceans.^1,2^

Some of the most studied biopolyesters include poly(lactic acid) (PLA), poly(butylene succinate) (PBS), and poly(butylene adipate-*co*-terephthalate) (PBAT), among many other polyesters and copolyesters.^3^ Nevertheless, ranking PLA, PBAT, and PBS in terms of their application weights can vary depending on the specific industry and context. PLA is commonly used in packaging materials, such as food containers, disposable cutlery, and biodegradable bags.^4^ It is also used in 3D printing filaments and biomedical applications such as sutures and drug delivery systems.^5,6^ PBAT is often found in compostable packaging films and bags, particularly for food packaging including the production of biodegradable shopping bags, disposable gloves, and agricultural mulch films, whereas PBS is commonly used in packaging films, particularly for food packaging and agricultural applications.^7−9^ It can also be found in compostable mulch films, disposable cutlery, and nonwoven textiles. While PLA, PBAT, and PBS all have significant applications in the biodegradable plastics market, their specific usage and market share can vary depending on factors such as regional regulations, technological advancements, and industry preferences.

However, the narrow processing window and brittleness of PLA and PBS generate the need to improve them. Many solutions can be done, such as the preparation of copolymers, blending with other polymers,^10^ or additives and fillers.^11^ In particular, the employment of PBAT and the preparation of binary blends with PLA and PBS turns up as a potential solution for making biopolyesters more resistant and with better properties. For instance, PLA/PBAT blends are widely employed for mulching films in agriculture,^10^ as well as other interesting applications, such as packaging, pharmaceutical or automotive industries.^12^ Nonetheless, the poor compatibility of PLA/PBAT^13^ and PLA/PBS binary blends^14^ encouraged the exploration of new approaches, such as the use of chain extenders or plasticizers, or the preparation of PLA/PBAT/PBS ternary blends, which has been reported to have excellent properties.^15,16^ Recently, some of us have prepared PLA/PBAT/PBS ternary blends fixing the PLA/PBAT ratio (1:1) and varying the amount of PBS. Analyzing the morphology, it was demonstrated that the PBS acts as a compatibilizer in the ternary blend, situating at the PLA/PBAT interface, reducing interfacial tension, preventing coalescence, thus refining the blend morphology and improving its mechanical properties.^17^

Many studies have reported interesting results regarding the biodegradation of these polyesters triggered by the action of externally added lipases. The vast majority of them are carried out under physiological conditions (i.e., 37 °C and pH 7–7.5).^18,19^ Huang et al. studied the enzymatic degradation of PCL, PBS, and PBSA films with different lipases in solution, reporting higher degradation for PCL and PBSA films.^20^ Lee et al. investigated the degradation mechanism of PBS under the action of a lipase from *Pseudomonas cepacia* by studying the degradation products obtained, revealing a surface etching mechanism in the enzymatic degradation of PBS.^21^ However, many factors influence enzymatic degradation: substrate (chemical and physical nature, size, molecular weight distribution, thermal properties), type of enzyme, media (nature of the solvent, pH), temperature, agitation, and moisture, among others.^22,23^ For instance, enzymatic degradation is enhanced in semicrystalline polymers with low molecular weight,^18^ under temperature conditions close to *T*_*m*_. This latter consideration might suppose a drawback for the self-stability of the enzymes, as high temperatures normally result in their denaturalization.^24^

*Candida antarctica* lipase B (CalB) is a widely employed enzyme for polymerization reactions (esterification) in organic solvents and depolymerization (hydrolysis of esters) in aqueous media.^25^ Previous studies have shown the best conditions for this lipase in terms of enzymatic activity, which is directly related to depolymerization and polymeric degradation: the optimum and most stable pH is in the range of 8–9 and temperature in the range of 35–40 °C.^26,27^ However, it remains stable from pH 3.5 to 9.5 in polar organic solvents and at temperatures as high as 150 °C.^28^ There are several studies in the literature regarding the enzymatic degradation of PLA, PBAT, and PBS, by externally adding CalB, which reported a wide range of results. Shinozaki et al. studied the enzymatic degradation of PLA films, obtaining a 50.4% degradation in 72 h.^29^ Kanwal et al. reported 15.7% weight loss in 12 days for PBAT films.^30^ In a recent publication from Hu et al., a ∼30% weight loss was observed after 35 days degradation of PBS films.^31^

Since the past decade, the novel approach of lipase-embedding into the polymer matrix has been developed as an alternative to externally added lipase degradation common tests.^32^ This embedded enzymatic degradation or self-degradation can have potential applications in agriculture, as many biopolyesters commonly exhibit limited environmental degradation.^33^ Enzyme embedding started with the preparation of CalB-embedded PCL films via the solvent-casting method from toluene, leading to complete degradation of the films in 24 h (for a CalB content of 6.5 wt %) and 17 days (1.6 wt %).^34^ Later, the same PCL films with 1.6 wt % CalB inside were studied under dynamic flow conditions, reducing the time required for total degradation.^35^ More recently, Jbilou et al. studied PBS degradation by reactive extrusion with CalB at 120 °C without any solvent involved. Results showed a high reduction in *M*_*n*_ (>90%) in 30 min.^36^ Iwasaki et al. obtained PLLA films with embedded Proteinase K by hot-pressing at 130 °C, reaching moderate weight loss after 1 week for a block copolymer containing PLLA.^37^

As many processing techniques are heat-dependent, recent studies have shown different approaches for increasing enzyme stability: chemical modification, enzyme immobilization, or including additives.^38^

In a recent publication from our group, alginate beads were employed to encapsulate a lipase from *Pseudomonas cepacia*. These particles maintained their activity even after heating them to 125 °C, which made obtaining PBS self-degradable films possible through melt-extrusion.^23^ In the past five years, other authors have successfully studied the self-degradation of several biopolyesters with different lipases. Huang et al. reported a ∼15% weight loss self-degradation of PLLA films by embedding an immobilized Proteinase K through melt extrusion and hot pressing at 200 °C.^38^ Four different lipases, including CalB, were successfully embedded through the same melt-extrusion/hot-pressing procedure, without further immobilization, into PBS (at 130 °C), PBSA (100 °C), and PCL (90 °C) matrixes, reaching significant degradation for PCL (100% degradation in 6 h) and PBSA films (100% degradation in 96 h) and moderate degradation for PBS films (20% in 504 h).^20^

To sum up, our research will deepen into the enzymatic self-degradation of three different biopolyesters—PLA, PBAT, and PBS—and their blends, addressed by the action of a lipase (CalB). A lipase-embedding process was followed to introduce CalB into the polyester matrix, turning them into self-degradable films via a melt-extrusion/hot-pressing procedure. This procedure applied on polymer blends, which has not been reported so far, will let us better comprehend the enzymatic degradation mechanisms of the studied biopolyesters, based on their different aliphatic/aromatic nature.

## 2 Materials and Methods

### 2.1 Materials

The following polymers were employed: a poly(lactic acid) (PLA) Ingeo 4043D grade (95–98% l-isomer according to the literature^39−48^) from NatureWorks Co. Ltd., USA; a poly(butylene adipate-*co*-terephthalate) (PBAT) Ecoflex F Blend C1200 from Polymats Co., Ltd., Thailand; and poly(butylene succinate) (PBS) BioPBSTM FZ91PM grade from PTT MCC Biochem Co., Ltd., Thailand. Molecular weight distribution and thermal properties are listed in Table 1. Three blend samples were prepared by melt blending in an internal mixer (Chareon Tut Co., Ltd., Thailand), where the mixing was performed at 190 °C with a rotor speed of 60 rpm for 10 min.^17^ The weight composition of each homopolymer in the blend was indicated with subscripts (the subscript after each homopolymer indicates the wt % of each polymer in the blend): PLA_50_PBAT_50_, PLA_45_PBAT_45_PBS_10_, and PLA_30_PBAT_30_PBS_40_. Lipase from *Candida sp.* recombinant, expressed in *Aspergillus niger* (CalB, ref L3170–50 ML), sodium phosphate monobasic monohydrate (ref S9638–1KG), and dibasic heptahydrate (ref S9390–1KG) for the preparation of the phosphate buffers (pH 7 and 8), *p*-nitrophenyl butyrate (pNPB, ref N9876–1G), dialysis membranes (SnakeSkin Dialysis Tubing 10,000 MWCO, ref 68100) were purchased from Sigma-Aldrich. Pluronic F-127 (ref P2443–250G) was acquired from Merck. Pluronic F-127 is a hydrophilic nonionic surfactant composed of a linear triblock copolymer from poly(ethylene oxide) (PEO) and poly(propylene oxide) (PPO). All of the reagents were used as received.

**Table 1:** Initial Molecular Weight Distribution (GPC) and Thermal Properties (DSC) from the Homopolymers Employed in This Study

	PLA		PBAT		PBS	
sample	pellet	film	pellet	film	pellet	film
*M*_*n*_ (x 10^4^)	8.98	9.22	3.77	4.16	4.28	5.06
*M*_*w*_ (x 10^4^)	17.78	18.00	10.16	10.97	17.76	18.07
*Đ*	2.0	2.0	2.7	2.6	4.2	3.6
*T*_*m*_ (°C)	152.0	149.8	120.2	120.0	113.9	112.5
*X*_*c*_ (%)	0.3	2.6	11.1	13.9	32.6	33.1

### 2.2 CalB Purification, Characterization, and Evaluation of the Enzymatic Activity

The extract from *Candida antarctica* lipase B (CalB, UniProt ID #LIPB_PSEA2), provided in liquid form, was washed following a typical methodology. First, several dialysis steps were performed employing the 10 kDa membrane in a phosphate buffer medium (10 mM, pH 7). Afterward, the liquid product was freeze-dried and preserved at −20 °C for further processes. Several batches were purified, employing 50 mL of the liquid lipase (reactant) and obtaining ∼1 g of the solid neat enzyme. Additionally, and as a way to prevent the denaturation of CalB due to the high temperatures reached during the processing, 1 g of Pluronic F-127 was dissolved in one batch of the dialyzed enzymatic solution in order to achieve a ∼ 50% w/w CalB/Pluronic ratio. Pluronic F-127 has been reported as a protecting agent for many lipases under high-temperature conditions or in acid/base media.^49,50^

Protein concentration was spectrophotometrically measured at 280 nm using a ε of 41,285 M^–1^·cm^–1^.^51^ The purity of the protein was assessed by sodium dodecyl sulfate-polyacrylamide gel electrophoresis (SDS-PAGE).

The lipase activity was evaluated through monitoring of the release of *p*-nitrophenol (pNP) from the hydrolysis of *p*-nitrophenyl butyrate (pNPB) at the maximum peak of absorbance of pNP, 410 nm, using a multimode plate reader.^22^ The substrate solution was prepared at 55.75 mM in absolute ethanol. In a 96-well plate, 2 μL of a lipase solution (0.20–0.25 mg/mL) were added to 200 μL of phosphate buffer solution (30 mM). Just before the measurement, 5 μL of pNPB substrate solution was added to each well, and the absorbance data were collected at 410 nm as a function of time. The experiments were performed at pH 7 and 8 (the media for our degradation studies) and 25 °C. All measurements were taken in triplicate. For the quantification of the enzymatic activity (*U*), the slope from the linear region of the UV curve (absorbance vs time; see Figure S1 in the Supporting Information, SI) was obtained for each replicate and employed in the following expression (eq 1).
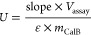
1where *U* stands for the enzymatic activity, the *slope* comes from the UV curves, the *V*_assay_ was fixed to be 207 μL, ε is the molar absorption coefficient −7447 M^–1^·cm^–1^ at pH 7 and 12,411 M^–1^·cm^–1^ at pH 8–, and *m*_CalB_ is the CalB mass (determined from the lipase concentration, the protein content, and the added volume of 2 μL).

### 2.3 Fabrication of Enzyme-Embedded Polyester Films

For the preparation of enzyme-embedded polyesters, the polyester powder (obtained by cryo-milling the pellets) and CalB powder were mixed at different CalB/polyester ratios (1, 5, and 10 wt %) and joint-extruded in a vertical double-screw MC 5 micro compounder (Xplore Instruments BV, The Netherlands). The extrusion process was performed at 125 °C for neat PBS with CalB and at 170 °C for the rest of the homopolyesters (i.e., PLA and PBAT) and blends. The CalB/polyester mixture was introduced in the micro compounder, mixed for 3 min at 60 rpm, and extruded at 60 rpm. Finally, the enzyme-embedded polyester films were prepared by hot-pressing the extrudates in a Collin P200E (Collin Solutions, GmbH, Germany) and Polystat 100T (Schwabenthan-Maschinen GmbH & Co. KG, Germany) pneumatic presses. The processing conditions consisted of four steps: (i) 50 s at 0 bar (preheating contact), (ii) 10 s at 5 bar, (iii) 30 s at 50 bar, (iv) 30 s at 100 bar, and (v) a final extra cooling step (at room temperature) for 30 min at 100 bar. Different temperatures were employed in steps 1–4 depending on the melting temperature of each polyester: 125 °C for PBS, 150 °C for PBAT, and 170 °C for PLA and the three blends.

As a way to protect CalB from the heating of the processing and its possible denaturation, a second set of samples was prepared through joint-extruding CalB/Pluronic (∼50 wt % CalB) and the different polyesters at 10 wt % of CalB/Pluronic with respect to polyester. The total amount of enzyme was 5 wt % in this case.

Samples were designated with their name, followed by the CalB content. For example, a film prepared using the PLA_50_PBAT_50_ blend and 5 wt % CalB/polyester will be referred to as *PLA*_*50*_*PBAT*_*50*_*_5%*. For those films prepared with Pluronic, the suffix *Plur* was added to the name of the sample (i.e., *PLA*_*50*_*PBAT*_*50*_*_5%Plur*).

The average thickness of all of the enzyme-embedded prepared films was 0.13 ± 0.03 mm, as determined by employing a digital caliper. In order to minimize possible denaturation of the enzyme and for better preservation of the films, the films were kept at 4 °C until the beginning of the experiments.

### 2.4 Analysis of the Weight Loss and Surface Morphology

Polymeric films (initial weight ∼10 mg, 8 × 8 mm^2^) were immersed individually into 4 mL glass vials containing 1 mL of phosphate buffer solution (24 mM, pH 8) and placed into a thermostated chamber (Stuart SI500 Shaking Incubator, Cole-Parmer, USA) at 40 °C with rotational stirring at 45 rpm. The samples were withdrawn at certain times, washed with distilled water, and finally weighed after complete drying at 50 °C (48 h). To control the enzymatic degradation, the weight loss of the samples was determined using the following eq (eq 2).
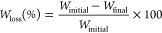
2where *W*_loss_ stands for weight loss (expressed in %), *W*_initial_ refers to the initial weight of the samples (before degradation), and *W*_final_ indicates the final weight of the samples after the degradation assays.

Morphological changes in the surface morphology and cross section of the films were monitored as a function of the degradation time through scanning electron microscopy (SEM) employing a Hitachi SU8000 scanning electron microscope (Hitachi, Ltd., Japan) and operating at 0.8 kV. Polyesters films were gold-coated (Polaron SC7640 Sputter Coater, Quorum Technologies, Ltd., UK) prior to the SEM analysis.

### 2.5 Analysis of the Changes in Molecular Weights and Chemical Structure in Degraded Samples and Liquid Aliquots

The number and weight-average molecular weights (*M*_*n*_ and *M*_*w*_, respectively) were determined through gel permeation chromatography analysis (GPC). Chromatograms were recorded in a Waters Instrument (Waters Corp., USA) equipped with RI and UV detectors. HR5E and HR2 Waters linear Styragel columns (7.8 × 300 mm, pore size 10^3^–10^4^ Å) packed with cross-linked polystyrene and protected with a precolumn were used. Samples were prepared by dissolving 3 mg of the sample in 1 mL of chloroform and using the same solvent as the eluent. Measurements were performed at 35 °C with a flow rate of 0.5 mL/min, and molecular weights were calculated against monodisperse polystyrene standards.

The chemical composition of the degraded films was also evaluated in a Nicolet iS20 FTIR spectrometer (Thermo Fisher Scientific, USA) equipped with a Smart iTX Accessory with a Diamond Crystal. Spectra were obtained from 4000 to 400 cm^–1^, at room temperature, with 32 scans and 4 cm^–1^ of resolution, and further analyzed with OMNIC software (v. 9.13.1294).

Finally, the liquid aliquots of each vial (containing 1 mL of phosphate buffer, CalB, and degradation products) were freeze-dried and further examined by proton nuclear magnetic resonance (^1^H NMR) in order to ascertain the possible products obtained during the degradation assays. Spectra were recorded in a Bruker AMX-300 spectrometer (Bruker Corp., USA). 640 scans were recorded from 10 mg sample solutions in 1 mL of deuterated water. Prior to the freeze-drying of the vials, pH measurements were performed in a pH 8+ DHS pHmeter (XS Instruments, Italy) to determine possible changes in the pH of the degradation media as a means to obtain further information about the enzymatic degradation mechanism of the biopolyesters under study.

### 2.6 Analysis of the Crystallinity as a Function of Degradation Time

The crystallinity of the films as a function of degradation time was evaluated by differential scanning calorimetry in a TA DSC25 equipped with an Intracooler RCS90 instrument (TA Instruments, Inc., USA). Temperature sweeps with two scans were done from −50 to 160 °C (PBS) or 180 °C (PLA, PBAT, and blends) at 10 °C/min. DSC curves were analyzed with TRIOS software (v5.7.0.56) from TA Instruments, Inc. For the analysis of the blend films, the heating scan was performed at 60 °C/min (to avoid PLA’s cold crystallization); however, it was impossible to differentiate the melting peaks of each homopolymers. For this reason, the melting enthalpy of the whole peak (or multiple peaks) was reported for the blends.

#### 2.6.1 Statistics

Data were used as generated with no preprocessing (e.g., transformation, normalization, evaluation/removal of outliers). Quantitative data are presented as the mean ± standard deviation in brackets. At least three independent replicates were evaluated for weight loss measurements.

## 3 Results and Discussion

An increasing number of recent publications in the literature report various enzymatic degradation studies on PLA, PBS, and PBAT under very different experimental conditions, which depend on the type of enzyme used. In fact, to the best of our knowledge, there are no standardized experiments regarding enzymatic degradation tests as in other degradation assays, such as soil burial or compost degradation. Results can vary significantly among studies for enzymatic degradation due to the different experimental conditions and the distinct nature of the starting polymers. Some parameters have been found to be determinants in the degradation of polyesters, such as the initial molecular weight distribution or the crystallinity.^52^ The influence of enzyme concentration has been studied in externally added enzyme tests as one of the multiple ways to control and tune the degradation.^53^ Herein, modulation of the enzyme-induced degradation of different polyester films through the preparation of self-degradable films is investigated.

### 3.1 Lipase Characterization and Evaluation of the Enzymatic Activity of CalB

As multiple degradation tests were performed with six different materials (three homopolyesters and three PLA/PBAT/PBS blends), several CalB batches were washed and freeze-dried. The *M*_*w*_ of the lipase under study, assessed through gel electrophoresis (SDS-PAGE), was found to be ∼33 kDa, in agreement with literature values.^54^ The protein content of each lyophilizate was evaluated by UV/vis (absorbance at 280 nm). Additionally, the enzymatic activity of each of the batches was evaluated at different pH values (7 and 8) as a way to select the best conditions for degradation tests; all of these parameters are included in Table 2.

**Table 2:** Description of the Protein Batches Used in This Work^b^

		*U* (μmol·s^–1^·mg^–1^)	
batch#	protein content (%) by UV_280_	pH 7	pH 8
1	47.8 (0.5)	0.2393 (0.0478)	0.2647 (0.0149)
2	43.7 (1.1)	0.2488 (0.0401)	0.3329 (0.0073)
3	47.0 (1.9)	0.1916 (0.0288)	0.2752 (0.0323)
Pluronic	37.9 (1.9)^a^	0.1537 (0.0280)	0.2243 (0.0263)

a Protein content was determined taken into consideration the 50/50 CalB/Pluronic ratio.

b CalB content of the lyophilizates was measured by UV at 280 nm, and the enzymatic activity was tracked by UV/vis. The standard deviation of the mean values is given in brackets.

In general, all the batches presented a protein content of 44–48%; however, the batch containing Pluronic showed a slightly lower protein content of ∼38%. Regarding the enzymatic activity, in all cases, CalB exhibited higher activity at pH 8 (10–46% higher, up to ∼91% in one of the batches); mean values were 0.2743 μmol·s^–1^·mg^–1^ at pH 8, and 0.2084 μmol·s^–1^·mg^–1^ at pH 7. These values were as expected, justifying the selection of this pH value for the degradation experiments due to the higher enzymatic activity at pH 8.^27^ Finally, the Pluronic batch presented slightly lower *U* values, probably due to the lower protein content. Similar observations are reported in the literature.^49^

### 3.2 Influence of CalB Content on the Self-Degradation of the Homopolymers: PLA, PBAT, and PBS

#### 3.2.1 Analysis of the Weight Loss and Morphological Characterization of the Degraded Samples

**Figure 1:**
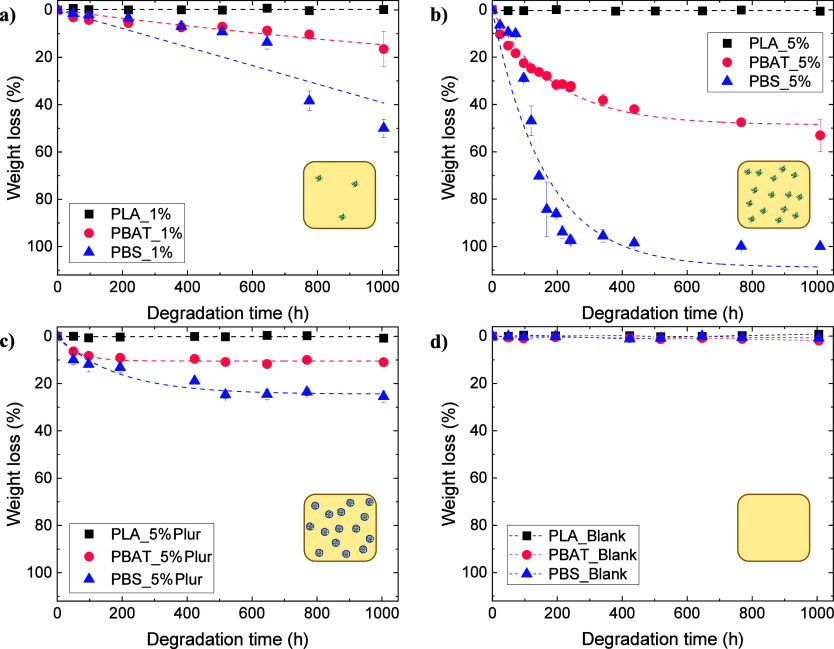
Weight loss curves for the three homopolyesters under study with increasing CalB/polyester ratio: (a) 1 wt % CalB (Polyester_1%), (b) 5 wt % CalB (Polyester_5%), (c) 5 wt % CalB/Pluronic (Polyester_5%Plur), and (d) blank experiments (without CalB). Dashed lines represent the fitting of the data to the different models: linear fitting (a), and the proposed model in eq 3 (b, c). Each graph includes a schematic representation of each system as an inset: the yellow square represents the polyester film, whereas the embedded lipase is plotted with an 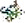
(embedded CalB) or 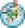 (embedded CalB/Pluronic). The symbols’ number is related to CalB content inside the films.

Figure 1 depicts the results corresponding to the weight loss of the three homopolymers under study with embedded CalB at two different concentrations, 1 and 5 wt % (Figure 1a,b, respectively). In Figure 1c, the results corresponding to weight loss of the 5 wt % CalB/Pluronic-embedded samples are shown for comparison purposes. Blank experiments, with neat polyester films in phosphate buffer solution (without CalB), were performed to study the hydrolytic degradation of the homopolymers, and the results in Figure 1d showed negligible weight loss.

The Polyester_1% films (i.e., 1 wt % CalB-embedded samples) exhibited moderate weight loss for PBS_1% films (∼50 wt % after 1000 h), with a lower extent in the case of the PBAT_1% films (∼16 wt % after 1000 h), as depicted in Figure 1a. When the CalB content inside the films increased from 1 to 5 wt % (Figure 1b), the trend changed from linear to exponential. In this case, PBAT_5% films reached ∼53 wt % weight loss by the end of the biodegradation test, whereas PBS_5% films exhibited complete degradation (100 wt % achieved). These results contrast with those obtained for the 5 wt % CalB/Pluronic films (Figure 1c), where PBAT_5%Plur films reached ∼11 wt % weight loss (4.5 times lower than in PBAT_5%), and ∼25 wt % for PBS_5%Plur films was obtained (∼4 times less than in PBS_5% samples). In all the cases, PLA films (i.e., PLA_1%, PLA_5%, and PLA_5%Plur) did not exhibit any weight loss (0–3 wt %). Weight loss results, together with the qualitative activity evaluation of the lipase-embedded films through the reaction with pNPB (and subsequent generation of pNP), suggested that added CalB inside the polyesters maintained its activity after melt extrusion at 170 °C without any further stabilization steps.

To quantify the extent of degradation and compare the different experiments, weight loss over degradation time curves were fitted to a previously employed model to describe the degradation kinetics quantitatively.^23^ This model, which is based on the Michaelis–Menten model for enzymes, describes the whole biodegradation process, which is controlled by heterogeneous reactions: (i) adsorption of the enzyme on the surface of the substrate, (ii) degradation of the polymer, and (iii) denaturation of the enzyme. Some authors have proposed a modified Michaelis–Menten model with some needed corrections for the enzymatic degradation of PLA that can be described by eq 3.^55,56^
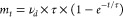
3where *m*_*t*_ refers to the weight loss of the sample (considered similar to *W*_loss_ indicated in eq 2), ν_*d*_ stands for the rate of degradation, τ indicates the rate of denaturation of the enzyme (time constant) and implies that the degradation has reached a *plateau*, and *t* is the time (expressed in hours). From the product between ν_*d*_ and τ, it is possible to obtain the value corresponding to the degradation at an infinite time (*A*). This model cannot be appropriately applied for cases where weight loss does not reach a plateau (as for example, in the case of the Polyester_1% experiments in Figure 1a). For these cases, only information regarding the rate of degradation will be obtained. A linear fitting is applied to avoid this issue, where the slope is associated with the degradation’s kinetics, and assumed to be similar to the degradation rate, ν_*d*_, in the modified Michaelis–Menten model (eq 3).

The application of the modified Michaelis–Menten model (eq 3) to the experimental data shown in Figure 1b,c allows us to extract further information on the effect of the CalB concentration, as well as the effect of Pluronic, on the self-degradation of the lipase-embedded homopolyester films (Table 3).

**Table 3:** Kinetic Parameters Determined from the Modified Michaelis–Menten Model for the Self-Degradation Studies in the Polyester_5% and Polyester_5%Plur Experiments, with the Three Homopolymers under Study (PLA, PBAT, and PBS)^b^

sample	CalB content (wt %)	ν_*d*_ (%/h)	τ (h)	*A* (%)	*R*^2^
PBAT_5%	5	0.2731 (0.0150)	171 (13)	46.7	0.9734
PBS_5%	5	0.6016 (0.0846)	175 (32)	105	0.9396
PBAT_5%Plur	5^a^	0.1744 (0.0269)	60 (10)	10.5	0.9586
PBS_5%Plur	5^a^	0.1342 (0.0258)	182 (42)	24.4	0.9303

a 50/50 CalB/Pluronic ratio.

b The standard deviation of the mean values is given in brackets.

It is important to note that, in all cases, PLA films exhibited a negligible weight loss (below 3 wt %); hence, fitting the experimental data to the modified Michaelis–Menten model was not feasible. The low weight loss exhibited by PLA films with embedded CalB was unexpected, as this lipase has been reported to degrade PLA successfully. Shinozaki et al. reported a ∼50% degradation for PLA cast films in 72 h.^29^ However, in a recent study from Rosato et al., CalB, as well as other enzymes, was unable to degrade PLA, whereas it successfully depolymerised other polyesters (e.g., PBSA and PCL).^57^ Several studies have demonstrated the preferential attack of proteases (e.g., Proteinase K) on PLLA, whereas PDLA enzymatic degradation is enhanced by the action of lipases, such as CalB.^57,58^ This explanation might be behind the negligible degradation observed for PLA films in our study, as PLA employed here has a high l-isomer. A second reason could be related to the initial molecular weight of PLA, which almost doubled those of the other two homopolyesters (Table 1), as well as the lower dispersity (*Đ*) of PLA films. Im et al. reported larger enzymatic degradation the lower the initial molecular weight of the PLA samples, evidencing the influence of this parameter on enzymatic degradation of polymers.^59^

Regarding PBAT and PBS films, experimental data in Figure 1 were successfully modeled with the Michaelis–Menten model, and the parameters are reported in Table 3. In the case of the 1 wt % CalB-embedded samples, no saturation in the weight loss curves was appreciated, similar to all PLA samples, and no plateau regime is reached (linear tendency), as shown in Figure 1a. The rate of degradation was found to be almost three times higher for the PBS films: 0.0415%/h (PBS_1%) > 0.0154%/h (PBAT_1%), meaning that PBS films were more degraded (according to the weight loss curves) than PBAT ones, under the selected conditions for CalB. In this particular case, the lower dispersity of PBAT films compared to that of PBS (see Table 1) could influence the enzymatic degradation of both polyesters.

Similar results were found when the CalB concentration was increased up to 5 wt % (Figure 1b), PBS films showed a 2-fold increase in the rate of degradation with respect to PBAT, being 0.6016%/h for PBS_5% and 0.2731%/h for PBAT_5%. On the other hand, the rate of denaturation of the lipase was found to be very similar for both homopolyesters: 171 and 175 h for PBAT_5% and PBS_5% films, respectively. This means that CalB denaturates or saturates at almost the same time. Finally, the degree of degradation at infinite time (*A*) was calculated to be ∼47 wt % for PBAT films and >100 wt % for PBS films. This 2-fold higher *A* value in the case of PBS_5% is attributed to the larger (double) degradation rate of these films. These degradation results are in accordance with other studies in the literature, which have shown improved degradation for PBS over PBAT, when degraded under the action of CalB.^60^ Although the initial molecular weight distribution of both homopolymers was similar, the initial degree of crystallinity was considerably higher for PBS than PBAT (33.1 vs 13.9%, respectively, as seen in Table 1), which would make PBAT films more prone to CalB degradation. Figure S2a in the SI shows the weight loss curve from the PBS_10% samples (i.e., films with 10 wt % CalB-embedded films), with comparable results with respect to PBS_5%: ν_*d*_ = 0.5872%/h for PBS_10% (0.6016%/h for PBS_5%), τ = 182 h for PBS_10% (175 h for PBS_5%), and *A* ≈ 100% in both cases. These observations show no enhancement in the overall degradation when the CalB content was increased from 5 to 10 wt % inside the PBS films, probably due to the high lipase content.

If we take a look at the kinetic parameters from the Polyester_5%Plur samples (Figure 1c), the incorporation of Pluronic led to a reduction in degradation when compared to PBAT_5% films, as evidenced by the lower values obtained for the kinetic parameters (from the modified Michaelis–Menten model): reduction of ∼36% in the rate of degradation, whereas the rate of denaturation dropped by ∼65%. On the other hand, PBS_5%Plur films showed a ∼78% reduction in ν_*d*_ compared to PBS_5% films, with no modification in τ: 182 h for PBS_5%Plur and 175 h for PBS_5%. This behavior means that enzyme encapsulation within Pluronic does not have the same effect on the enzymatic degradation of PBS films as compared to PBAT films as it delays the degradation by affecting only ν_*d*_ and not τ, as in the case of PBAT_5%Plur samples (where Pluronic affects both parameters). The lower weight loss exhibited by the 5 wt % CalB/Pluronic-embedded films could be related to the lower enzymatic activity exhibited by the CalB when encapsulated within Pluronic, as shown in Table 2. This encapsulation with Pluronic was done for heat-protecting the enzyme; however, this procedure possibly decreases the contact between the enzyme and polymer, thus exhibiting a lower degradation rate.

The values of the reported kinetic parameters in Table 3 for PBAT and PBS are in the same order of magnitude as reported in the literature for PCL degraded by the action of a lipase from *P. cepacia* (ν_*d*_ = 0.004–0.156%/h, τ = 27–586 h),^56^ and PLA films with Proteinase K (ν_*d*_ = 0.95–1.96%/h, τ = 41–98 h).^55^

The images of the degraded films are included in Figure S3 in the SI.

**Figure 2:**
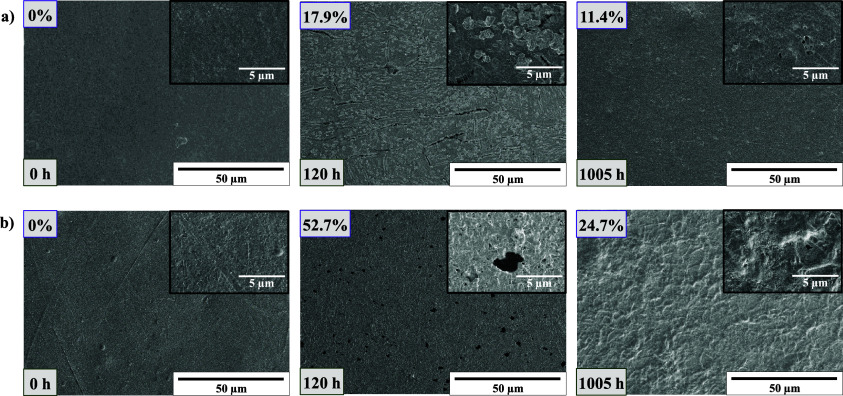
Scanning electron microscopy images taken at 20× magnification for 5 wt % CalB-embedded polyester films: (a) PBAT (from left to right: control film; PBAT_5% and PBAT_5%Plur) and (b) PBS (from left to right: control film; PBS_5% and PBS_5%Plur). In the upper left corner, the achieved weight loss is shown, whereas the degradation time (in hours [h]) is included in the lower left corner. The insets show images at a higher magnification for easier visualization of the extent of degradation.

Scanning electron microscopy was employed to ascertain the surface morphology of degraded 5 wt % CalB embedded polyester films, PBS, and PBAT after 120 h, those being the samples that showed major changes on their weight loss as shown in Figure 1. For comparison, SEM images corresponding to degraded 5 wt % CalB/Pluronic-embedded films after 1005 h degradation are also shown (shorter times showed nonappreciable weight loss). As observed in Figure 2 (images on the center), both 5 wt % embedded CalB films, PBAT_5% and PBS_5%, showed the appearance of small holes after 120 h with respect to control films (images on the left) being those much more evident for PBS films. Interestingly, SEM images taken at a higher magnification for PBAT_5% after 120 h (inset center Figure 2a) revealed the formation of agglomerates of material on the surface of the sample, which might indicate the occurrence of surface erosion mechanism; such a feature is not observed for the sample PBS_5% after 120 h (inset center Figure 2b). SEM images corresponding to the cross section of samples PBAT_5% and PBS_5% are shown in Figure S4 in the SI. The sample PBAT_5% did not reveal major changes in morphology with respect to the control sample, in agreement with the occurrence of a surface erosion degradation mechanism. In contrast, the sample PBS_5% showed the presence of holes on the inside of PBS films, which might indicate a bulk erosion degradation mechanism for this sample. Further experiments presented later on will help elucidate the degradation mechanism.

For 5 wt % CalB/Pluronic-embedded films (Figure 2, images on the right), some possible degraded areas in the form of small holes can be observed after 1005 h. However, the extent of degradation is much smaller compared to 5 wt % embedded CalB films, in agreement with the lower weight losses achieved after 120 h (it was not possible to obtain SEM images of PBAT_5% and PBS_5% films after 1005 h degradation). Additionally, it is important to note that CalB does not degrade Pluronic, a PEO-based derivative, and a migration of the Pluronic through the film toward the surface could also be possible. This hypothesis is aligned with the visual observations in Figure 2.

#### 3.2.2 Physicochemical Characterization of the Degraded Samples and Liquid Aliquots

**Figure 3:**
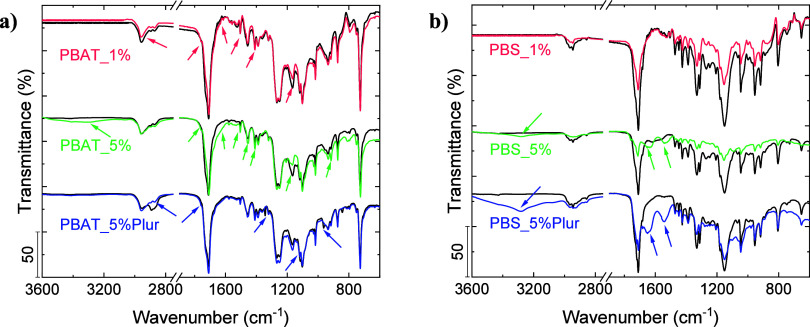
FTIR spectra corresponding to (a) PBAT and (b) PBS. Nondegraded (black) and degraded films (after 1000 h) for the 1 wt % CalB-embedded films (red), 5 wt % CalB-embedded films (green), and 5 wt % CalB/Pluronic-embedded films (blue).

The chemical composition of the films after degradation was monitored through FTIR spectroscopy, and the results are shown in Figure 3. Regarding PLA films, representative peaks are located at ∼1749 cm^–1^ and assigned to the C=O stretching from the ester group; at ∼1081 cm^–1^, attributed to the C–O stretching of secondary alcohol generated in the hydrolysis; at ∼1178 cm^–1^, assigned to the C–O stretching from the ester group; at ∼1451 cm^–1^ corresponding to the CH_2_ vibration; and at ∼2996 and ∼2945 cm^–1^, assigned to the CH asymmetric stretching.^10,61^ No appreciable changes are observed between the FTIR spectra corresponding to the pristine PLA films and those corresponding to degraded samples, regardless of enzyme concentration (1 and 5 wt %) or the presence of Pluronic for enzyme encapsulation (Figure S5 in the SI). This is in agreement with the negligible weight losses observed in Figure 1 for PLA films.

In the case of PBAT films (Figure 3a), the FTIR spectra corresponding to PBAT_1% degraded films showed a decrease in the intensity from the characteristic peak of the C–O stretching of the ester group, from the esterification of a primary alcohol with adipic or terephthalic acid (1165 cm^–1^) with respect to that corresponding to the nondegraded film, thus confirming the breakage of the ester bonds.^62^ Additionally, the appearance of bands in the FTIR spectra corresponding to the degraded film located at 1410 cm^–1^ corresponding to CH_2_ angular deformation band, at ∼1650 cm^–1^ corresponding to the C=O stretching of COOH end groups (from the degradation products), and the stretching peak of free C=O groups (1755 cm^–1^) further confirmed the degradation.^1^ PBAT_5% films exhibited higher variations in the bands; the peak at ∼3300 cm^–1^ was assigned to OH stretching, which additionally confirmed the degradation of PBAT films. On the other hand, PBAT_5%Plur films presented lower changes in FTIR spectra; the most noticeable is the decrease of the intensity of the band located at ∼2895 cm^–1^, which could be related to the partial solubilization of Pluronic and also confirmed the degradation.^30^ There are other bands that showed a slight increase in intensity, as marked in Figure 3 with arrows.

Finally, the FTIR spectra corresponding to degraded PBS films showed a significant decrease in the band intensity of all peaks with respect to that of pristine PBS films (Figure 3b). As in the case of PBAT, the appearance of new bands in the spectra corresponding to the degraded sample located at ∼3286 and ∼1652 cm^–1^ assigned to OH stretching and C=O stretching of COOH end groups (from the degradation products) additionally confirmed the degradation, as well as the broad band due to hydrogen bonding. It is important to note that for the spectra of the degraded PBS films, the appearance of a peak at ∼1546 cm^–1^ that can be assigned to the amide II band of CalB N–H deformation and C–N stretching further confirmed the degradation of PBS films, consistent with the exposure of CalB toward the surface of the films.

**Figure 4:**
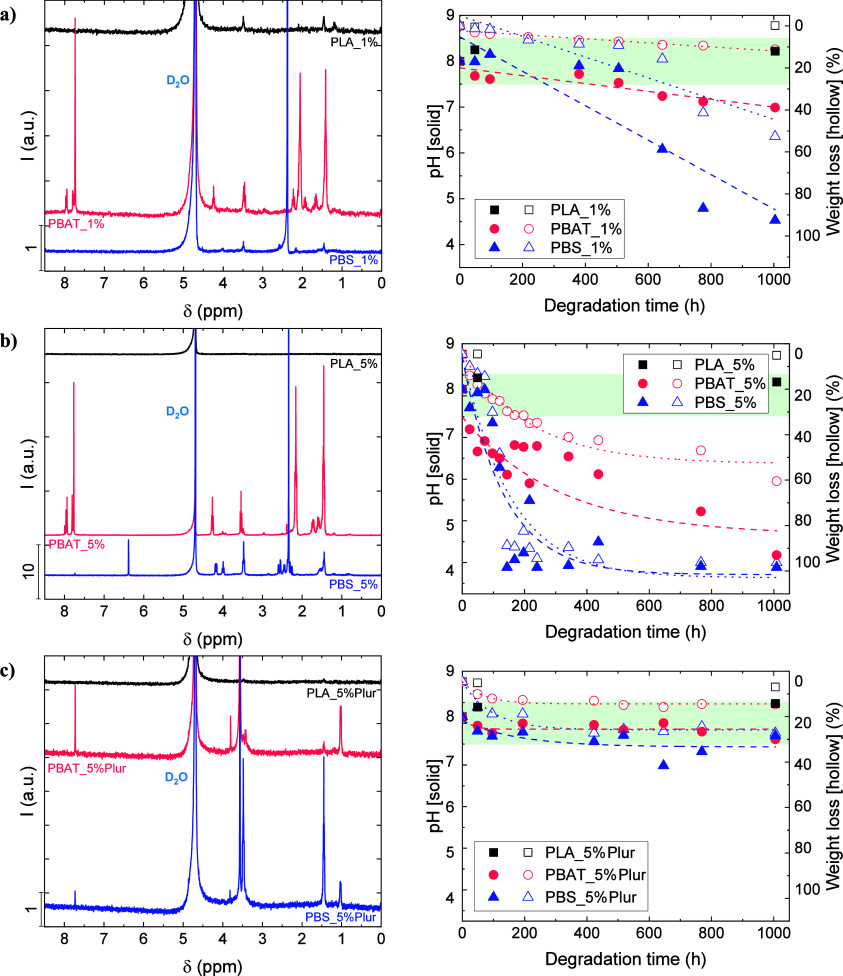
^1^H NMR spectra (left) and variation in pH (right) from the liquid aliquots of (a) 1 wt % CalB-embedded experiments, (b) 5 wt % CalB-embedded experiments, and (c) 5 wt % CalB/Pluronic-embedded experiments. In the pH variation graphs on the right (solid symbols), weight loss curves (hollow symbols) have been added for comparison purposes. The green area represents the pH value of the employed phosphate buffer (pH 8 ± 0.5).

The appearance of degradation products as a result of enzymatic degradation of the film was further confirmed by ^1^H NMR and analysis of the pH from the degradation media (Figure 4). The detection of the different monomers in the liquid aliquots suggests an endo-type degradation for CalB.^61^

In the case of PLA_1%, the ^1^H NMR spectrum exhibited the characteristic signals of lactic acid (1.46, and 3.49 ppm), as evidenced in Figure 4a, left. However, its low concentration (deduced from the low intensity of the peaks) did not have an effect on pH, as it remained constant at ∼8 (Figure 4a, right). On the other hand, ^1^H NMR spectra from the PBAT_1% and PBS_1% experiments (Figure 4a, left) showed much higher intensity for the characteristic peaks of the degradation products: 1.66 and 3.48 ppm (butanediol), 1.42 and 2.06 ppm (adipic acid), and 7.78 and 7.96 ppm (terephthalic acid) in the PBAT_1% assays; and small peaks from butanediol (1.46 and 3.49 ppm) and an intense peak from succinic acid (2.38 ppm) in the case of PBS_1%. The generation of these subproducts agreed with the pH of the degradation media, which was significantly reduced, as seen in Figure 4a, right: final pH values were ∼7 (PBAT_1%) and ∼4.5 (PBS_1%). This acidification process is attributed to the generation of degradation products, mainly acids, with p*K*_a_ values of 3.54 (terephthalic acid), 3.86 (lactic acid), 4.21 (succinic acid), and 4.41 (adipic acid).^63^ The lower acidification observed in PBAT_1% might be attributed to a lower generation of subproducts, as indicated by the 10-fold lower intensity of the ^1^H NMR peaks with respect to PBS_1% and the lower weight loss (Figure 1). Similar observations were obtained in a previous study with PBAT films, where enzymatic degradation by the employment of CalB slightly reduced the pH of the media.^30^

When CalB content was increased from 1 to 5 wt %, the signals from PBAT and PBS degradation subproducts were detected at a higher concentration (intensity is ten times higher than in the previous case), as evidenced in Figure 4b, left. These results are in accordance with the achieved weight loss (Figure 1), which was significantly larger, and the pH of the degradation media also confirmed the growth in concentration of the subproducts: pH values dropped to ∼4 in PBAT_5% and PBS_5% (Figure 4b, right). As in previous experiments, PLA_5% did not exhibit a pH change by the end of the assay, as almost no lactic acid residues were detected from ^1^H NMR spectra.

The incorporation of Pluronic to CalB led to lower degradation, which was confirmed by ^1^H NMR spectra, as depicted from the lower intensity peaks in Figure 4c, left, with no detection of adipic or succinic acids. These results are supported by the negligible pH variation maintained in the range of the studies (pH 8 ± 0.5), as appreciated in Figure 4c, right. The most intense peak in PBAT_5%Plur and PBS_5%Plur was detected at 3.58 ppm and assigned to PEG from Pluronic.

#### 3.2.3 Analysis of Changes in Molecular Weight Distribution over Time

**Figure 5:**
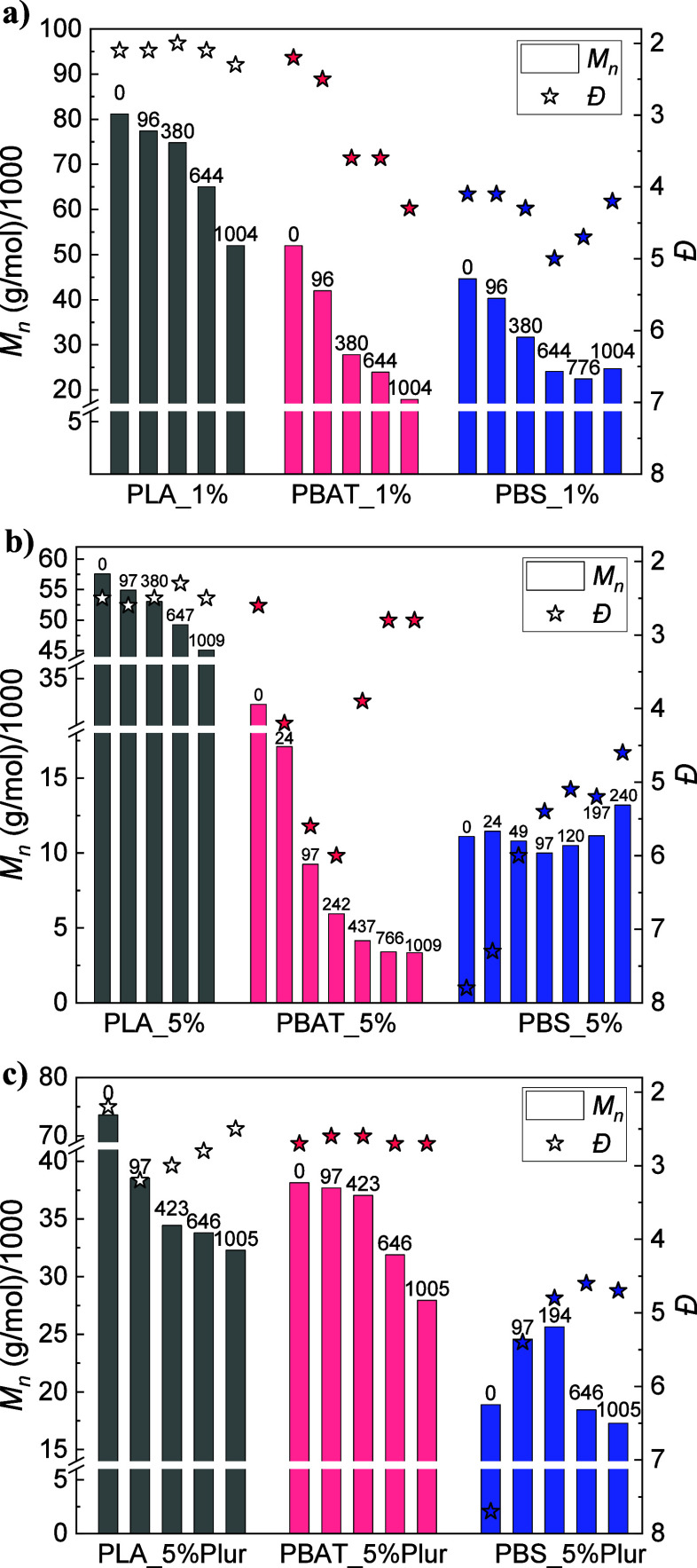
Molecular weight distribution (GPC) for the three homopolymers in (a) 1 wt % CalB-embedded films, (b) 5 wt % CalB-embedded films, and (c) 5 wt % CalB/Pluronic-embedded films. Degradation times (in hours) are indicated at the top of each bar.

The molecular weight distribution over the degradation time was monitored in degraded samples through GPC. GPC results from the control experiments (blank) are included in Figure S6 in the SI. In the case of the polyesters with embedded 1 wt % CalB (Figure 5a), there is a noticeable decrease in *M*_*n*_, for the three samples under study. PLA_1% showed a decrease of ∼36% (Đ maintained constant at ∼2), whereas a ∼66% decrease was observed for PBAT_1% (*Đ* increased from ∼2 to ∼4) and PBS_1% exhibited a ∼ 45% decrease (*Đ* first increased from ∼4 to ∼5, tending to ∼4 by the end of degradation). A similar observation for PBS has been reported previously in the literature, in a study in which a PBS film exhibited a significant decrease in *M*_*n*_ (from 45,000 to 14 000 g/mol), whereas *M*_*w*_ maintained almost constant, making *Đ* increase from 1.5 to 4.1.^64^ For PLA films, the moderate decrease in *M*_*n*_ and the fact that *Đ* does not change in the degraded samples with respect to pristine films suggests a moderate hydrolytic degradation of the films, which is reflected in a very low amount of weight loss (Figure 1). In contrast, for PBAT and PBS, the larger changes in *M*_*n*_ observed for degraded samples, together with the noticeable increase in dispersity, are consistent with a mechanism of enzymatic degradation induced by the action of CalB. This lipase commonly acts by cutting the long polymeric chains into shorter chains, a reason that can explain the high reduction in *M*_*n*_ and increase in *Đ*.^61^

When CalB content was increased from 1 to 5 wt %, *M*_*n*_ in PLA films was not varied as much, as evidenced by Figure 5b: PLA_5% showed a reduction of ∼22% (*Đ* maintained constant at ∼2.5), and PBS_5% films, which exhibited total degradation in Figure 1b, did not present any change in *M*_*n*_. Surprisingly, *Đ* was reduced from ∼8 to ∼4.5 at 240 h. On the contrary, PBAT_5% reached an ∼90% decrease in *M*_*n*_, up to values as low as 3000 g/mol (*Đ* remained constant at ∼3, with a sharp increase to ∼6 in the medium-range degradation times). Such a reduction in *Đ* can be attributed to the low *M*_*n*_ achieved at the end of degradation, making CalB cut the long chains that still remain in the polymer. Xu et al. reported a reduction in *M*_*n*_ for PBAT samples, decreasing from ∼30,000 to ∼10 000 g/mol,^65^ similar to that observed for PBAT_1% and PBAT_5% films. These observations for PBAT_5% and PBS_5% will be commented on later on.

Finally, GPC results corresponding to the sample with incorporated Pluronic (Figure 5c) showed a much higher reduction of *M*_*n*_ in PLA_5%Plur, which was ∼56%, although weight loss was null. This disagreement in degradation might be explained by the systematic cutoff of long polymeric chains into shorter ones, with no erosion on the films. On the other hand, the other two homopolymers (i.e., PBAT and PBS) presented a lower reduction in *M*_*n*_: ∼27% (PBAT_5%Plur, which showed a ∼11 wt % weight loss) and ∼8% (PBS_5%Plur, with a ∼ 25 wt % weight loss). Dispersity (*Đ*) was maintained constant at ∼2.5 for both PLA_5%Plur and PBAT_5%Plur films, with a reduction from ∼8 to ∼4.5 in 1000 h for PBS_5%Plur films. These results could be related to their lower degradation, as shown by the weight loss experiments depicted in Figure 1.

The behavior in PBS films with 5 wt % CalB (both alone and with Pluronic) is quite atypical and contrasted to PBS_1% films (with a more pronounced reduction in *M*_*n*_ and an increase in *Đ,* as seen in Figure 5a), as *Đ* usually increases in enzymatic degradation assays, at the same time that *M*_*n*_ gets reduced.^35^ The presence of a higher amount of CalB could have had a hydrolytic degradation effect on PBS, as reported in previous research, where a drastic decrease in *M*_*n*_ was found after extrusion of PBS in the presence of CalB.^36^ PBS films suffered a significant decrease in the initial *M*_*n*_ from the reference films without embedded lipase: ∼12% in PBS_1%, and ∼78 and ∼63% for PBS_5% and PBS_5%Plur, respectively. Moreover, this effect was also appreciated for the other two studied homopolymers (i.e., PLA and PBAT) to a lower extent. For instance, PLA films suffered a reduction in *M*_*n*_ of ∼12% when adding 1 wt % CalB, ∼38% in PLA_5%, and ∼20% in PLA_5%Plur. In the case of PBAT films, initial *M*_*n*_ was not reduced in PBAT_1% (∼25% increase) and diminished by ∼17% and ∼8% for PBAT_5% and PBAT_5%Plur, respectively. The lower reduction observed in PBAT films suggests a higher resistance of this polymer toward degradation by CalB, possibly due to the presence of the terephthalate groups.^22^ Hence, GPC results from PBS_5% (Figure 5b) and PBS_5%Plur (Figure 5c) are in line with the fact that only short segments are removed from the end-chains, making *M*_*w*_ diminish while keeping *M*_*n*_ constant, and thus, reducing *Đ*. This generally happens when the enzyme reaches the crystalline regions of the polymer, and it is only observed in PBS films due to the higher crystallinity of this polyester (as depicted from Table 1),^57^ and the larger initial degradation (higher reduction in initial *M*_*n*_), possibly due to the degradation during the extrusion process.^36^ Nonetheless, note that *Đ* in PBAT_5% films started to diminish from ∼6 to ∼2 in the medium-range degradation times (Figure 5b), in a way similar to that in PBS_5% films.

All these observations in PBS films are in accordance with a bulk erosion mechanism, as optical micrographs revealed that the films maintained the original dimensions (at low degradation times), and the appearance of holes was also appreciated, together with a reduction in the molecular weight.^66^ In the case of PBAT films, the decrease in *M*_*n*_ observed by GPC would also suggest a bulk erosion mechanism, despite the absence of holes in the PBAT films, which are more related to a surface erosion mechanism, as reported in previous studies.^67^ The embedding of CalB inside the polyester films might be behind the change in the erosion mechanism,^68^ which has been reported to enhance both surface and bulk erosion.^69^

#### 3.2.4 Analysis of the Crystallinity of the Degraded Samples

Previous studies in the literature have ascertained the occurrence of degradation in the case of PLA, PBAT, and PBS through analysis of the crystallinity. In the case of PBAT samples, Xie et al. showed a small reduction from 9.5 to 5.7%.^67^ Shi et al. reported a small reduction in crystallinity in the case of PBS films, from 57 to 49%.^61^ Enzymes in general, and CalB in particular, usually start degrading the amorphous regions of the films, and through this process, the shorter newly generated chains from the amorphous domains can crystallize and, thus, increase the degree of crystallinity.^61^

**Figure 6:**
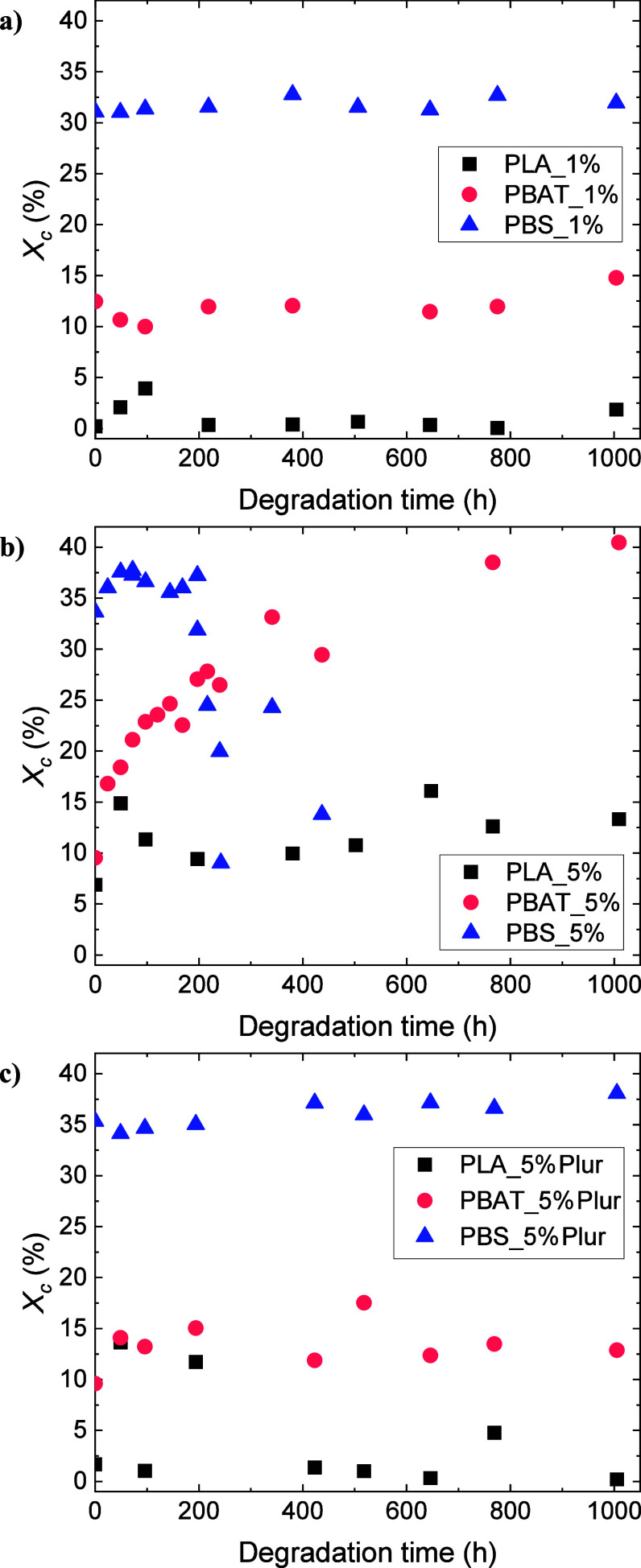
Changes in the degree of crystallinity (*X*_c_) of the degraded samples: (a) 1 wt % CalB-embedded experiments, (b) 5 wt % CalB-embedded experiments, and (c) 5 wt % CalB/Pluronic-embedded experiments.

The first heating (DSC) of the degraded samples was used for the evaluation of the initial, intermediate, and final values for the crystallinity of the films (Figure 6). The degree of crystallinity (*X*_*c*_) of the homopolymers was determined from the melting enthalpy (Δ*H*_m_), in the first heating scan, of the peak of each homopolymer, and the equilibrium melting enthalpy (Δ*H*_m_^0^) of each of the homopolymers, which was considered to be 93 J/g for PLA,^61^ 114 J/g for PBAT,^70^ and 213 J/g for PBS,^71^ as detailed in eq 4.
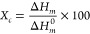
4

DSC results revealed no significant variation in crystallinity in the experiments carried out with the lowest CalB content (i.e., 1 wt % CalB), as appreciated in Figure 6a. Regarding PLA_1% films, *X*_*c*_ maintained almost invariant in the range 0–5%, whereas in PBAT_1% and PBS_1%, the crystallinity values remained stable at ∼12 and ∼32%, respectively. Surprisingly, when CalB content was increased from 1 to 5 wt % (Figure 6b), PLA_5% samples exhibited a higher initial *X*_*c*_ than previously observed (∼7%), with a growing trend by the end of the experiment (∼13%). PBAT_5% films showed a large increase in *X*_*c*_, from ∼10 to ∼40%, which could be related to a selective attack on the amorphous regions of PBAT, making the crystalline/amorphous ratio higher.^30^ Similar behavior has been reported with PBAT mulching films in different degradation conditions and ascribed to the faster degradation in amorphous regions by the action of microorganisms and enzymes.^72^ This latter result contrasted with PBS_5% films, which showed a decreasing tendency in *X*_*c*_, from values of ∼35 to ∼10%. At the very first stages of degradation, the degree of crystallinity experienced a slight increase, which could be related to a recrystallization process. However, when degradation speeds up, the enzyme also attacks the crystalline region of PBS, which is why *X*_*c*_ displays such a reduction.^61^

Incorporating Pluronic into the films had a clear effect on the crystallinity, as PBAT_5%Plur and PBS_5%Plur samples did not exhibit any variation in the degree of crystallinity, as appreciated in Figure 6c. These results could be supported due to the lower degradation achieved. The slight increase observed in the initial *X*_*c*_ of PLA_5%, PBS_5%, and PBS_5%Plur films could also be explained by the degradation during extrusion due to the higher amount of CalB.^36^

### 3.3 Self-Degradation in Polymer Blends

**Figure 7:**
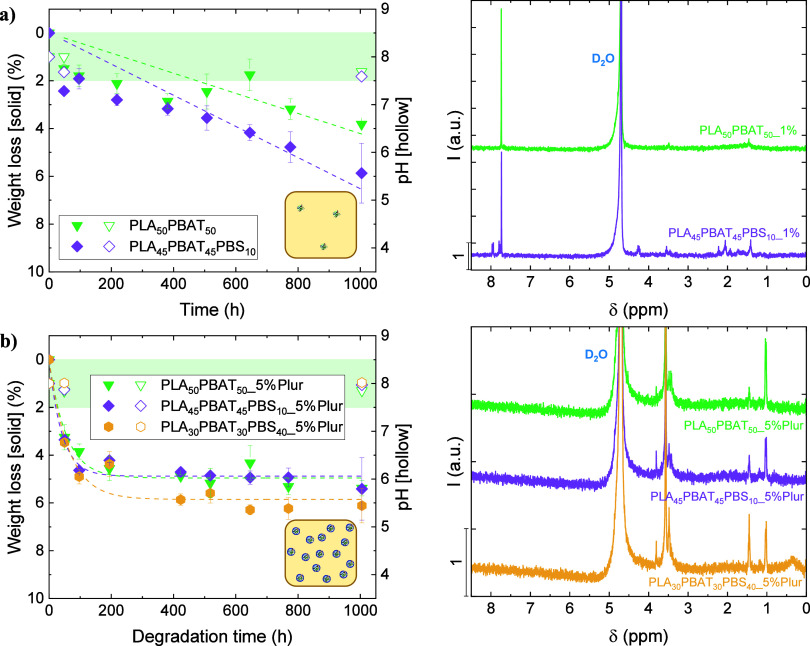
Weight loss curves for the three blends under study (solid symbols) and variation in pH of the aliquots from the degradation media (hollow symbols) in left figures, and ^1^H NMR spectra obtained from the liquid aliquots from the degradation media (right figures): (a) 1 wt % CalB-embedded films, and (b) 5 wt % CalB/Pluronic-embedded films. A schematic representation of each system is included as an inset in the left graphs: the yellow square represents the polyester film, whereas the embedded lipase is plotted with an 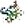
(embedded CalB) or an 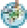
(embedded CalB/Pluronic). The symbol number is related to CalB content inside the films. The green area represents the pH value of the employed phosphate buffer (pH 8 ± 0.5).

After studying the enzymatic self-degradation of homopolymers (i.e., PLA, PBAT, and PBS), a similar study was carried out for blends of these materials. These blends are materials with potential application for biodegradable packaging and hence the interest in them.^17^ The study of the weight loss of the 1 wt % CalB-embedded films revealed very low degradation in all the blends (see Figure 7a, left), which were slightly higher than that exhibited by PLA_1%. When Pluronic was incorporated into the films, no change was observed from the weight loss curves (Figure 7b, left), showing similar results for all the blends and in the same range as 1 wt % CalB-embedded samples (4–6 wt % weight loss).

The weight loss curves were further fitted with the modified Michaelis–Menten model, and the results are included in Table 4. In this case, a 21-fold and 17-fold increase in degradation rate was obtained for PLA_50_PBAT_50__5%Plur and PLA_45_PBAT_45_PBS_10__5%Plur films, respectively, with regard to the degradation kinetics from the 1 wt % CalB-embedded films (i.e., PLA_50_PBAT_50__1% and PLA_45_PBAT_45_PBS_10__1%, respectively), determined from the slope of the linear fitting of the weight loss data. The ν_*d*_ data from the blends presented lower values with regard to that obtained for the homopolymers PBAT_5%Plur and PBS_5%Plur (see Table 3). Nevertheless, the low rate of denaturation of the lipase (∼50 h) resulted in a similar degradation at infinite time (*A*) compared to 1 wt % CalB-embedded films: ∼ 5 wt % for PLA_50_PBAT_50__5%Plur and PLA_45_PBAT_45_PBS_10__5%Plur, and ∼6 wt % for PLA_30_PBAT_30_PBS_40__5%Plur films. In both lipase-embedding systems depicted in Figure 7, the polyester blends showed the same behavior: the higher the PBS content, the faster the degradation kinetics, and the higher the weight loss achieved.

**Table 4:** Kinetic Parameters Determined from the Modified Michaelis–Menten Model for the Self-Degradation Studies in the 5 wt % CalB/Pluronic-Embedded Films, with the Three Blends under Study^d^

sample	CalB content (wt %)	ν_*d*_ (%/h)	τ (h)	*A* (%)	*R*^2^
PLA_50_PBAT_50__1%	1	0.0042 (0.0007)^a^	n.d.^b^	n.d.^b^	0.8498
PLA_45_PBAT_45_PBS_10__1%	1	0.0065 (0.0007)^a^	n.d.^b^	n.d.^b^	0.9206
PLA_30_PBAT_30_PBS_40__1%	1	n.a.	n.a.	n.a.	n.a.
PLA_50_PBAT_50__5%Plur	5^c^	0.0902 (0.0145)	55 (9)	5.0	0.9573
PLA_45_PBAT_45_PBS_10__5%Plur	5^c^	0.1082 (0.0206)	45 (9)	4.9	0.9680
PLA_30_PBAT_30_PBS_40__5%Plur	5^c^	0.1037 (0.0086)	58 (5)	6.0	0.9899

a Obtained from the linear fit of the data.

b These parameters could not be obtained, as the weight loss curves were linearly fitted due to low weight loss exhibited by the films from the blends with higher content in PLA.

c 50/50 CalB/Pluronic ratio.

d The standard deviation of the mean values is given in brackets.

As for the case of the homopolymers, the pH of the degradation media was analyzed as a function of time, and the results showed that no changes in pH were detected, as depicted in Figure 7a,b (left). Analysis of the chemical composition of the aliquots from degradation media through ^1^H NMR experiments showed the characteristic peak of terephthalic COOH groups (7.74 ppm) in both samples from 1 wt % CalB-embedded films (Figure 7a, right), as well as lactic acid (1.46 and 3.49 ppm) in PLA_50_PBAT_50__1% and adipic acid (1.42 and 2.06 ppm), butanediol (3.46 and 4.28 ppm), succinic acid (2.23 ppm), and lactic acid (3.55 ppm) in the PLA_45_PBAT_45_PBS_10__1% sample, with much lower intensity. On the contrary, with the incorporation of Pluronic into the films, it was not possible to appreciate the characteristic peaks of the degradation subproducts; only PEG signals from Pluronic were detected, as depicted in Figure 7b, right. Degradation results from the analysis of the films’ weight loss from the blends, as well as ^1^H NMR from the liquid aliquots, evidenced minor variations among the studied blends with slightly higher degradation compared to PLA films, which presented the lowest degradation due to its nature (predominant l-isomer). In the studied blends, the main components are PLA and PBAT at equal ratios in the three blends (50, 45, and 30 wt %), which could possibly explain the low degradation exhibited by the self-degradable films obtained from the blends.

The chemical composition of the films prepared from the blends before and after degradation (1000 h) was evaluated through FTIR, and results in Figure S7 (in the SI) evidence the low degradation of these films. The most relevant change in the degraded films was the appearance of the stretching vibration of free C=O groups (∼1756 cm^–1^). A slight reduction in the intensity of the peaks was appreciated in some samples, in accordance with the low weight loss appreciated in Figure 7, following the trend of the previously commented PLA samples.

**Figure 8:**
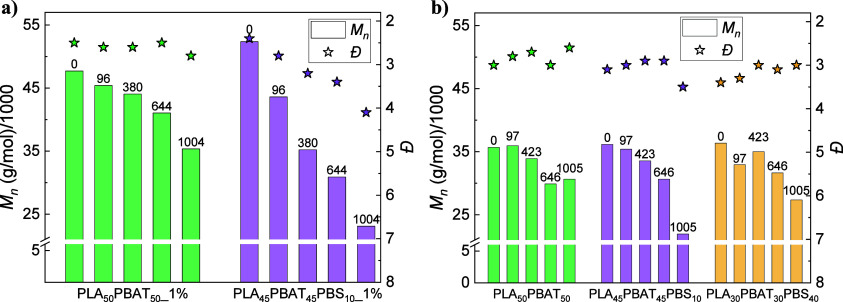
Molecular weight distribution (GPC) for the three blends under study: (a) 1 wt % CalB-embedded films and (b) 5 wt % CalB/Pluronic-embedded films. Degradation time (in hours) is indicated at the top of each bar in GPC graphs (top).

The degradation of the films was further studied through the analysis of the evolution of the molecular weight distribution over the degradation time. GPC results shown in Figure 8 revealed a significant reduction in *M*_*n*_ of the two blends under study. In the case of the samples tested with 1 wt % CalB inside the films (Figure 8a), there is a noticeable decrease in *M*_*n*_: ∼25% in PLA_50_PBAT_50__1% and ∼56% in PLA_45_PBAT_45_PBS_10__1%. When Pluronic was incorporated into the 5 wt % CalB/Pluronic-embedded films, the reduction in *M*_*n*_ was not as high as before (Figure 8b): ∼14% for PLA_50_PBAT_50__5%Plur films, ∼39% for PLA_45_PBAT_45_PBS_10__5%Plur samples, and ∼25% for PLA_30_PBAT_30_PBS_40__5%Plur films.

The dispersity, on the other hand, was maintained constant in the 2.5–3 range; only the PLA_45_PBAT_45_PBS_10_ blends, PLA_45_PBAT_45_PBS_10__1%, and PLA_45_PBAT_45_PBS_10__5%Plur showed significant increases of *Đ* up to values of ∼4 and ∼3.5, respectively. The low or negligible weight loss appreciated in many samples (Figure 7), as well as a moderate reduction in *M*_*n*_ (Figure 8) could be related to hydrolytic degradation of the films. The higher degradation in PLA_45_PBAT_45_PBS_10__1% and PLA_45_PBAT_45_PBS_10__5%Plur samples (slightly higher weight loss in Figure 7) could be related to the presence of PBS in this blend, as this homopolymer was found to achieve a higher degradation. These results would support a hydrolytic degradation mechanism induced by the catalytic action of CalB.

First heating (DSC) of the degraded samples revealed no variation in crystallinity for the blends (Figure S8 in the SI). In this case, it was not possible to distinguish the melting of PLA and PBS (narrow and well-defined peaks) from the wide PBAT melting peak; hence, the total melting enthalpy (Δ*H*_*m*,total_) was reported instead. In general terms, Δ*H*_*m*,total_ kept stable during the whole degradation experiment, with a slight increase in the PLA_45_PBAT_45_PBS_10_ samples: from ∼7 to ∼13 J/g (PLA_45_PBAT_45_PBS_10__1% in Figure S8a), and from ∼13 to ∼17 J/g (PLA_45_PBAT_45_PBS_10__5%Plur in Figure S8b). Regarding PLA_50_PBAT_50_ samples, the total melting enthalpy increased from ∼5 to ∼10 J/g when Pluronic was added to 5 wt % CalB/Pluronic-embedded films (Figure S8b).

## 4 Conclusions

This study conducted a thorough comparative investigation about the mechanism of degradation of self-degradable polyester films prepared by embedding a lipase (*Candida antarctica* lipase B, CalB) within PLA, PBAT, and PBS. The results showed that the degradation of PBAT and PBS CalB-embedded films was greatly enhanced by the increase of the CalB content inside the films (from 1 to 5 wt %). Furthermore, the incorporation of Pluronic F-127 to CalB evidenced no real need to protect the lipase from thermal denaturation, as this study also demonstrated that lipases incorporated into these polymers maintained their activities after melt extrusion at 170 °C without any further stabilization steps. Additionally, results from CalB/Pluronic-embedded films also supported this hypothesis: the weight loss was about 4–5 times lower when compared to films with similar content in CalB. The encapsulation of CalB within Pluronic is used as a common heat-protecting procedure; nevertheless, the encapsulation possibly decreases the contact between CalB and the polyester films and thus lowers the degradation rate. Regarding the nature of the polyesters, CalB showed a higher preference toward PBS, as evidenced by the kinetics obtained from the modified Michaelis–Menten model applied to weight loss curves: PBS > PBAT > PLA. PLA films did not show any degradation for all of the tested conditions, which is in accordance with the high l-isomer content.

The characterization of the degraded samples revealed further information regarding the degradation mechanism of CalB. The increase in crystallinity of PBAT together with scanning electron microscopy observations suggests that the mechanism of self-degradation for this homopolymer is based on a surface erosion mechanism, as shown by the appearance of agglomerates of the material on the surface of degraded PBAT films. However, the lipase-embedding procedure might have modified the degradation mechanism of PBAT films, as revealed by GPC, which suggests a bulk erosion mechanism due to the decrease in *M*_*n*_. Possibly, both mechanisms are happening in PBAT degradation. PBS seems to be degraded through autocatalyzed hydrolysis, which results in drastic changes in crystallinity, surface morphology, FTIR data, and weight loss curves, which support a bulk erosion mechanism of CalB on this polyester.

Finally, the lipase-embedding procedure was further applied to blends from these three polyesters with increasing PBS composition. Degradation results evidenced minor variations among the studied blends, with slightly higher degradation compared with PLA films. The main components of the polymer blends under study are PLA and PBAT at equal ratios in the three blends (50, 45, and 30 wt %). Therefore, modest degradation was observed due to the nature of PLA’s l-isomer. ^1^H NMR analysis carried out in the liquid aliquots further supported the lack of degradation of PLA in the blends, as only terephthalic acid from PBAT was detected as a subproduct. However, the addition of PBS (10 wt %) to the second blend enhanced its degradation, possibly because of the faster degradation of PBS in the presence of CalB, as demonstrated by the higher *M*_*n*_ reduction. The studied lipase-embedding procedure exhibited promising results for PBS, as well as for PBAT films, when the CalB content was increased. Nonetheless, the low effectiveness of the self-degradation of PLA, together with the blends, exposed the need to select the most suitable enzyme for degrading each biopolyester or all the components in blends. This study is a proof-of-concept for the development of plastic materials with enhanced degradation capabilities in well-known processes, such as compost-based degradation. A future approach for enzymatic degradation of enzymes could lie in finding a common enzyme that degrades all the components in the blend or in exploring a mixture of enzymes.

To sum up, this research has shed more light on the enzymatic degradation of several polyesters, and in more detail, it has deepened into the self-degradation of three biopolyesters and their blends achieved through lipase-embedding.

## Supporting Information Available

Example for the determination of the enyzmatic activity of CalB, self-degradation results for PBS_10% films, visual aspect of degraded films, SEM images of the cross-section of PBAT_5% and PBS_5% films, FTIR spectra of nondegraded and degraded PLA films, molecular weight distribution through GPC for the blank experiments, FTIR spectra of nondegraded and degraded films obtained from the three blends, and changes in the degree of crystallinity of the degraded samples from the three blends (PDF)
