# Supplementary material for: Enzymatic Degradation Behavior of Self-Degradable Lipase-Embedded Aliphatic and Aromatic Polyesters and Their Blends
Source: Biomacromolecules. 2024 Jun 10;25(7):4030–45. doi: 10.1021/acs.biomac.4c00161 (PMC11238343; doi:10.1021/acs.biomac.4c00161)
Supplement: Supplementary file 1 — bm4c00161_si_001.pdf [file bm4c00161_si_001.pdf]

Supplementary Information

Enzymatic degradation behavior of self-degradable lipase-embedded aliphatic and aromatic polyesters and their blends

Mario Iván Peñas,[a,b] Ana Beloqui,[c,d] Antxon Martínez de Ilarduya,[e] Supakij Suttiruengwong,[f] Rebeca Hernández,[a] Alejandro J. Müller[b,d]**

**Corresponding authors: rhernandez@ictp.csic.es, alejandrojesus.muller@ehu.es*

[a] Institute of Polymer Science and Technology ICTP-CSIC, Juan de la Cierva 3, 28006 Madrid, Spain

[b] Polymat and Department of Polymers and Advanced Materials: Physics, Chemistry and Technology, Faculty of Chemistry, University of the Basque Country UPV/EHU, Paseo Manuel de Lardizabal 3, 20018 Donostia-San Sebastián, Spain

[c] Polymat and Department of Applied Chemistry, Faculty of Chemistry, University of the Basque Country UPV/EHU, Paseo Manuel de Lardizabal 3, 20018 Donostia-San Sebastián, Spain

[d] IKERBASQUE, Basque Foundation for Science, Plaza Euskadi 5, 48009 Bilbao, Spain

[e] Department of Chemical Engineering, Polytechnic University of Catalonia ETSEIB-UPC, Diagonal 647, 08028 Barcelona, Spain

[f] Sustainable Materials Laboratory, Department of Materials Science and Engineering, Faculty of Engineering and Industrial Technology, Silpakorn University, 73000 Nakhon Pathom, Thailand

Figure S1. Determination of the enzymatic activity from the UV curve at 410 nm.

Figure S2. Weight loss curve and optical micrographs taken at 20x magnification for the PBS_10% films.

Figure S3. Visual aspect of the degraded films for the different experiments.

Figure S4. SEM images corresponding to the cross-section of PBAT_5% and PBS_5% films.

Figure S5. FTIR spectra of non-degraded and degraded PLA films (after 1000 h) for the three different systems.

Figure S6. Molecular weight distribution (GPC) of the degraded films from the blank experiments.

Figure S7. FTIR spectra of non-degraded and degraded films (after 1000 h) obtained from the three blends for the two systems under study.

Figure S8. Changes in the degree of crystallinity (X_c) of the degraded samples from the three blends for the two systems under study.

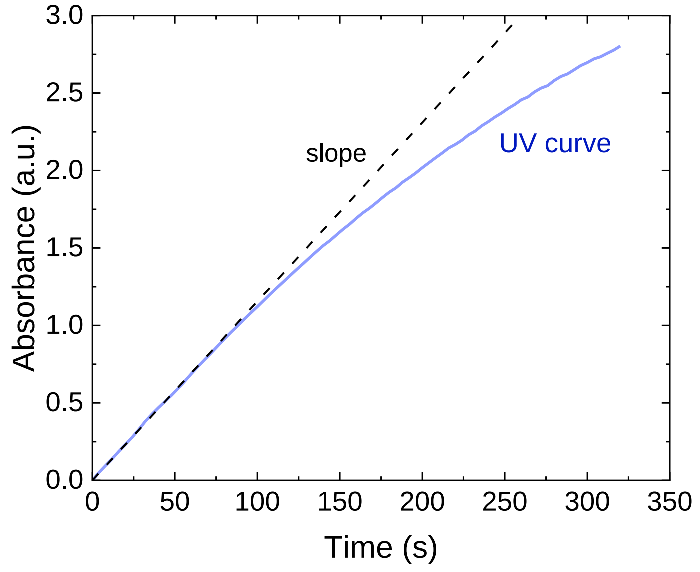

Figure S1. UV curve employed for the determination of the enzymatic activity of CalB. The slope has been determined from the linear region (initial region) of the UV curve (absorbance at 410 nm vs. time).

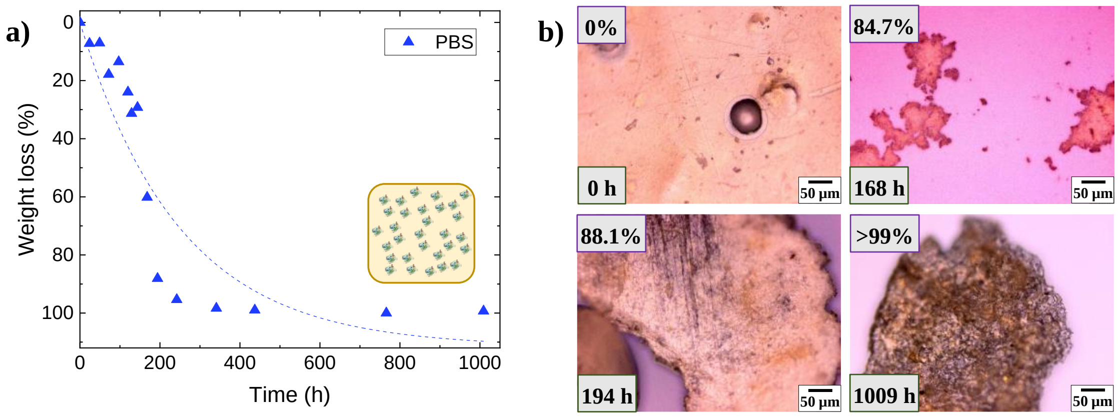

Figure S2. a) Weight loss curve for the PBS_10% films. Dashed lines represent the fitting of the data to the proposed model in Equation 3. A schematic representation of the system is included as an inset in the graph: the yellow square represents the polyester film, whereas the embedded CalB is plotted with. Symbols' number is related to CalB content inside the films. **b)** Optical micrographs taken at 20x magnification for the PBS_10% films. In the upper left corner, the achieved weight loss is shown, whereas the degradation time (in hours [h]) is included in the lower left corner.

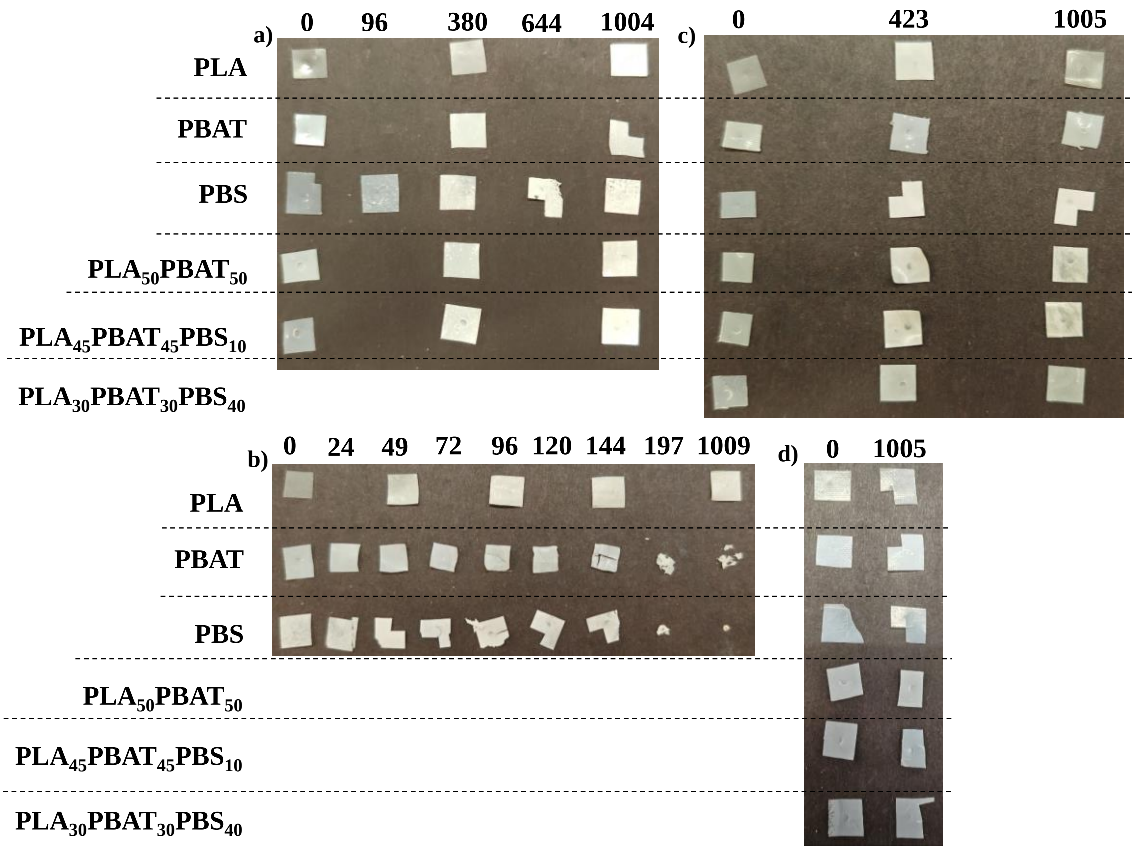

Figure S3. Visual aspect of the degraded films for the different experiments: **a)** 1 wt% CalB-embedded films, **b)** 5 wt% CalB-embedded films, **c)** 5 wt% CalB/Pluronic-embedded films, and **d)** blank experiments. The degradation time (in hours) is included at the top.

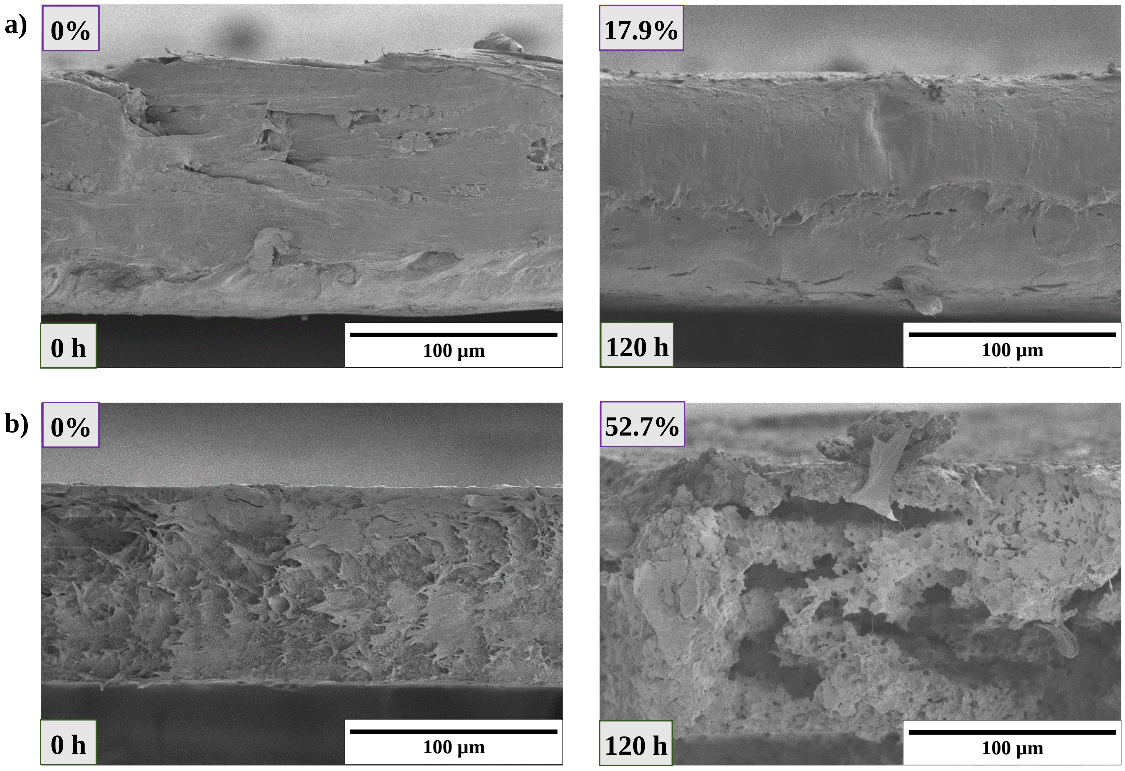

Figure S4. SEM images taken at 20x magnification corresponding to the cross-section of **a)** PBAT (from left to right: control film and PBAT_5%) and **b)** PBS (from left to right: control film and PBS_5%) films. In the upper left corner, the achieved weight loss is shown, whereas the degradation time (in hours [h]) is included in the lower left corner.

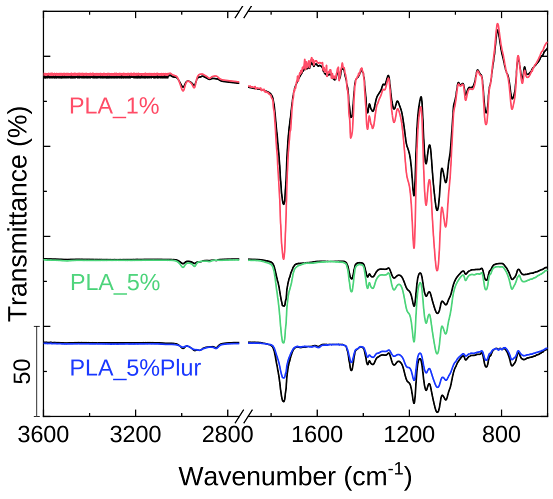

Figure S5. FTIR spectra of non-degraded (in black) and degraded PLA films (after 1000 h) for the 1 wt% CalB-embedded films (red), 5 wt% CalB-embedded films (green), and 5 wt% CalB/Pluronic-embedded films (blue).

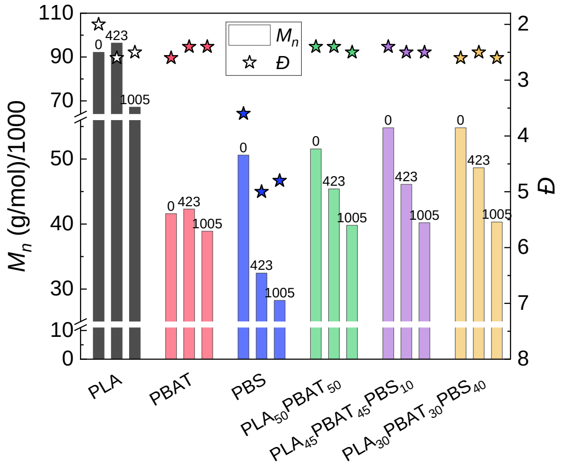

Figure S6. Molecular weight distribution (GPC) of the degraded films from the blank experiments. Degradation time (in hours) is indicated in the top of each column.

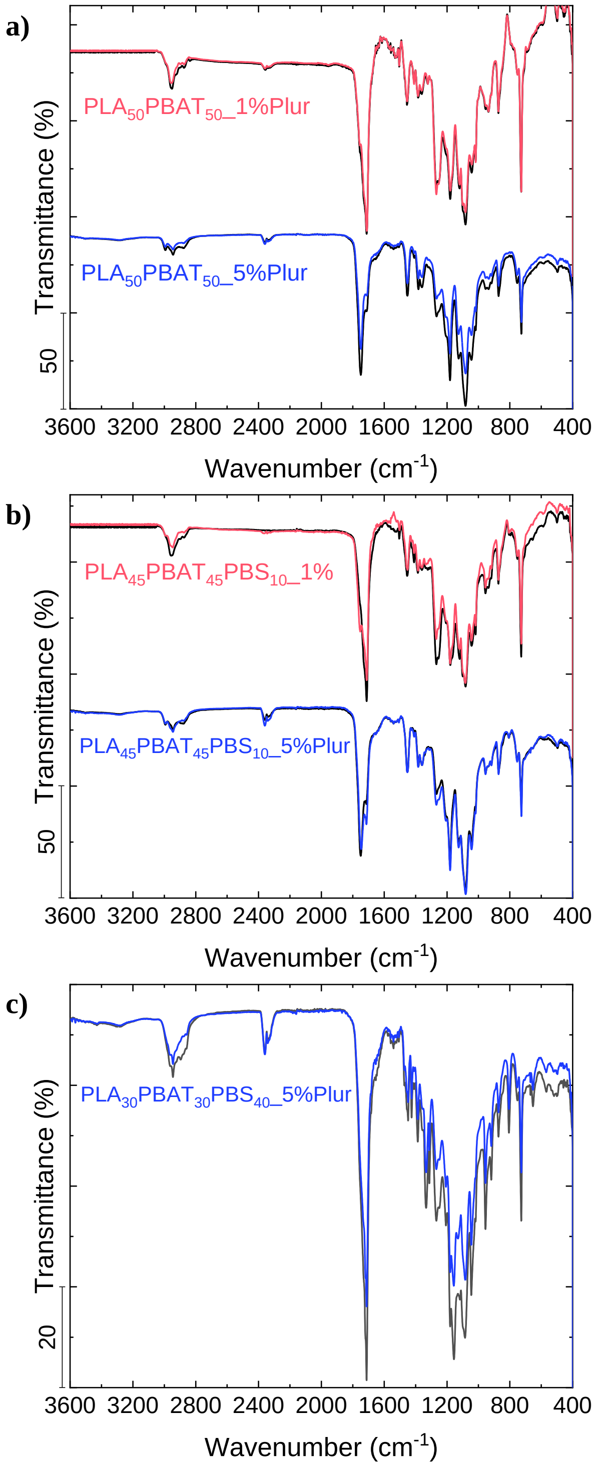

Figure S7. FTIR spectra of non-degraded (in black) and degraded films (after 1000 h) obtained from the three blends for the 1 wt% CalB-embedded films (red), and 5 wt% CalB/Pluronic-embedded films (blue): **a)** $PLA_{50}PBAT_{50}$, **b)** $PLA_{45}PBAT_{45}PBS_{10}$, and **c)** $PLA_{30}PBAT_{30}PBS_{40}$.

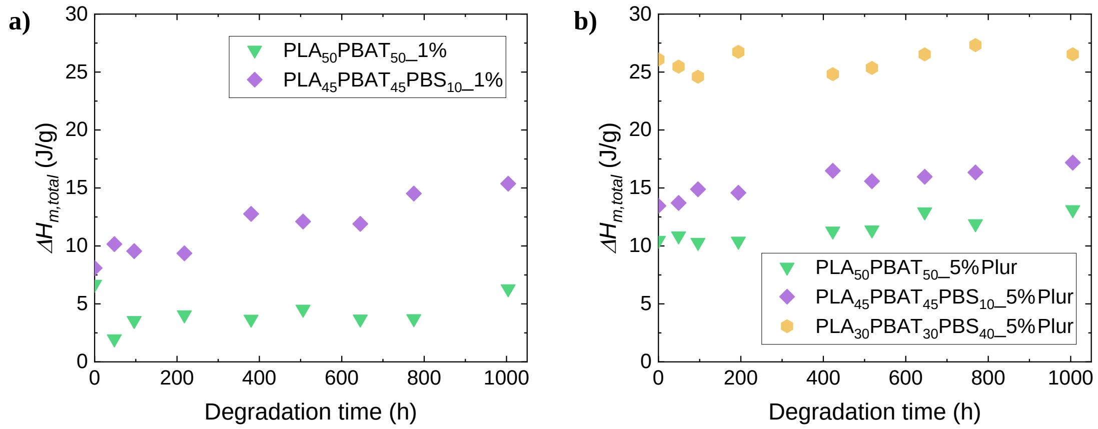

Figure S8. Changes in the degree of crystallinity (X_c) of the degraded samples from the three blends under study: **a)** 1 wt% CalB-embedded experiments, and **b)** 5 wt% CalB/Pluronic-embedded experiments.
